# Implementation and Assessment of a Curriculum for Renal Point of Care Ultrasound (POCUS) Training 

**DOI:** 10.24908/pocus.v9i2.17479

**Published:** 2024-11-15

**Authors:** Yoshiko Ishisaka, Hong Yu Wang, Hayato Mitaka, Elliot Charen, Paru Patrawalla

**Affiliations:** 1 Department of Medicine, Icahn School of Medicine at Mount Sinai, Mount Sinai Morningside and West New York, NY USA; 2 Department of Medicine, Division of Allergy and Infectious Diseases, University of Washington Seattle, WA USA; 3 Division of Nephrology, Icahn School of Medicine at Mount Sinai, Mount Sinai Beth Israel New York, NY USA; 4 Division of Pulmonary and Critical Care Medicine, Icahn School of Medicine at Mount Sinai, Mount Sinai Morningside and West New York, NY USA

**Keywords:** Renal Ultrasound, Point-Of-Care Ultrasound, POCUS Education, Internal Medicine

## Abstract

**Purpose:** Renal ultrasound is a non-invasive method to assess for obstructive acute kidney injury (AKI). Point of care ultrasound (POCUS) has been shown to be a good screening tool for obstructive AKI, and with formal training, has high sensitivity and specificity. We aimed to evaluate the effectiveness and feasibility of integrating a novel renal POCUS curriculum into an existing two-week nephrology rotation for internal medicine residents.** Methods:** We enrolled internal medicine residents rotating on a two-week nephrology rotation between September 2022 and June 2023. Pre-recorded online lectures and a hands-on session on image acquisition were provided. Pre-and post-rotation confidence questionnaires and knowledge tests were collected. At the end of the rotation, participants were evaluated using a skills checklist. Evaluation for knowledge retention was assessed 6–12-months post-rotation with a post-survey and knowledge test.** Results:** Of the 16 residents that were enrolled, 12 residents completed pre- and post-rotation questionnaires and tests, and 15 residents completed the 6–12-month follow-up. The confidence level showed significant improvement post-test and at 6–12-month follow-up. Knowledge test scores showed a trend towards improvement that did not achieve statistical significance (pre- 6.0 [5.0-7.25], post- 6.5 [5.75-8.0], 6–12-months 7.0 [6.0-8.0] p=0.40). On the skills checklist, an average of 16.8 out of 18 steps were done correctly. **Conclusion:** Our study showed confidence improvement and a trend towards knowledge improvement after integrating a novel Renal POCUS curriculum into a nephrology rotation. Further iterative changes, such as deliberate practice, or practice with immediate feedback, should be considered.

## Introduction

Renal ultrasound is a recommended, non-invasive method to assess for obstructive etiologies of acute kidney injury (AKI) 1, which can be seen in 1-10% of patients with AKI 2, 3. Traditionally, consultative ultrasound (i.e. technician performed and radiology interpreted ultrasound) is ordered as part of the evaluation of AKI in the hospital. However, with the increased availability of point of care ultrasound (POCUS), consultative ultrasound increases costs and delay care 4. In fact, in our institution, there was a two-day delay from the time a renal ultrasound was ordered to obtaining the final radiology read 5. 

Renal POCUS can be performed at the bedside and has been shown to be a quick and reliable method to detect obstructive AKI by trained physicians 6, 7. These findings have led the Society of Hospital Medicine to recommend the use of Renal POCUS for the diagnosis of AKI in inpatients 8. However, there is limited evidence on how to train internal medicine residents in renal POCUS and concerns about adding to an already compressed and complex residency workflow and curriculum.

We describe the development of a renal POCUS curriculum that was fully integrated into an existing two-week nephrology rotation for internal medicine residents to minimize exacerbating a hectic workflow and to maximize clinical relevance and expert supervision. This quality improvement project assessed the impact of this curriculum on learner confidence, knowledge, and skills in renal POCUS. 

## Methods

### Study Design

This is a retrospective review of de-identified data collected from residents who completed a nephrology rotation at Mount Sinai Beth Israel between September 2022 and June 2023. The Mount Sinai Beth Israel Quality Improvement Committee approved this study and internal medicine residents who underwent this novel POCUS curriculum agreed to participate in the quality evaluation.

### Study Population

Internal medicine residents were assigned to a two-week nephrology rotation during their second year. The Renal POCUS curriculum was integrated into the nephrology rotation beginning in September 2022 for all residents assigned to the rotation. Residents were encouraged to watch pre-recorded lectures on basics of ultrasonography and Renal POCUS prior to the start of their rotation. They attended a bedside hands-on session on Renal POCUS image acquisition at the start of their rotation. Our strategy of curriculum development based on Kern’s six-step model.

**Table 1:** Our strategy of curriculum development based on Kern’s six-step model.

**Step**	**Strategy**
Problem identification and general needs assessment	Literature review -Radiology-read renal ultrasonography is often unnecessary and leads to increased healthcare costs -Trained physicians can reliably and quickly perform renal POCUS -Retrospective chart review at our institution showed unnecessary radiology-read renal ultrasounds performed and a two-day delay from when it was ordered
Needs assessment of targeted learners	Internal medicine resident survey - Low confidence in identifying hydronephrosis on ultrasound Review with residency program and nephrology leadership - Existing two-week nephrology elective where residents evaluate patients with AKI -Existing expertise of nephrology attendings in renal POCUS - Busy consult workflow limiting availability of nephrology attendings to perform renal POCUS studies
Goals and specific objectives	- Improve confidence and knowledge of hydronephrosis identification on ultrasound - Practice image acquisition skills for renal POCUS - Integrate renal POCUS into the workflow of evaluating a new patient with AKI
Educational strategies	- Asynchronous didactic resources, including reading material and an e-lecture on ultrasound basics and renal POCUS - Hands-on training on image acquisition - Portfolio of exams done and reviewed with faculty
Implementation	- Shared a link to asynchronous material prior to the first day of their elective - Scheduled time at the beginning of their elective to teach image acquisition skills for renal POCUS at the bedside - Requested residents to maintain a procedure log
Evaluation and feedback	- Comparison of pre-, immediately post- and 6-12-months post-elective confidence and knowledge in Renal POCUS - Post-elective image acquisition skills using a checklist - Feedback for iterative improvement

### Curriculum Development

We designed our curriculum based on Kern’s six-step model for curricular development 9. We summarized our approach to these six steps in Table 1. For step one, we performed a literature review to identify the problem of a possible overreliance on radiology-read ultrasound and the utility of physician-performed renal POCUS. Additionally, we performed a retrospective chart review and found that there were unnecessary renal ultrasound exams performed and a two-day delay in radiology-read renal ultrasonography at our institution 5. For the needs assessment of targeted learners (step 2), we identified that learners had low confidence in their ability to identify hydronephrosis. In addition, nephrology attendings had expertise in renal POCUS but limited time to perform indicated studies due to a busy workflow. Residency program leadership identified an existing two-week nephrology rotation that was required for all residents, highlighting the need to integrate any new curriculum within this timeframe. The goals and objectives of this curriculum (step 3) were the following: improve confidence and knowledge of hydronephrosis identification on ultrasound; practice image acquisition skills for renal POCUS; and integrate renal POCUS into the workflow of evaluating a new patient with AKI. The educational strategies used (step 4) were the following: use of asynchronous didactic resources, including reading material and an e-lecture on ultrasound basics and renal POCUS; and hands-on training in image acquisition and maintenance of a portfolio of practice exams done and reviewed with faculty. For the implementation of this curriculum (step 5) with each resident scheduled for the nephrology rotation, we shared a link to the asynchronous material prior to the first day of their elective; scheduled time at the beginning of their rotation to teach image acquisition skills for renal POCUS at the bedside; and requested residents to maintain a procedure log. For the final step of evaluation and feedback, we compared pre-, immediately post-, and 6–12-months post-rotation confidence and knowledge in Renal POCUS; post-rotation image acquisition skills using a checklist; and informal feedback for iterative improvement.

### Learner Assessment

A survey was administered to assess learner confidence pre-rotation, immediately post-rotation, and 6–12-months post-rotation. Survey questions regarding self-confidence and skills were rated on a numerical scale of 1-5, with 5 representing high confidence. Residents also completed image-based multiple-choice tests pre-rotation, immediately post-rotation, and 6–12-months post-rotation to assess their knowledge of Renal POCUS. The 10-question knowledge test included content on ultrasound basics, and normal and abnormal findings of the kidney and bladder. The pre-and immediately post-rotation 

questions were identical. The 6-12-month follow-up questions included the same content with modification of the stems and images. At the end of the rotation, the learners’ image acquisition skills were evaluated using an 18-item behavior-based checklist. Residents were encouraged to perform POCUS on new nephrology consults for AKI under the supervision of the nephrology attending. The number of acquired POCUS images was directly reported to us at the time of the post-rotation questionnaire, and the range of the number of POCUS images (increments of five) that each participant obtained in the next 6–12-months after the elective was also reported at the time of the 6–12-month questionnaire. Whenever available, the POCUS images were compared with the radiology-performed ultrasound reading. 

### Statistical Analysis

All statistical analyses were done on R studio 2022.12.0+353. T-test was done when the results were numerical continuous values in a normal distribution, and Wilcoxon’s test was performed in non-normal distribution. A chi-square test was done for categorical values. The p-values were considered significant at the level of <0.05.

**Figure 1:**
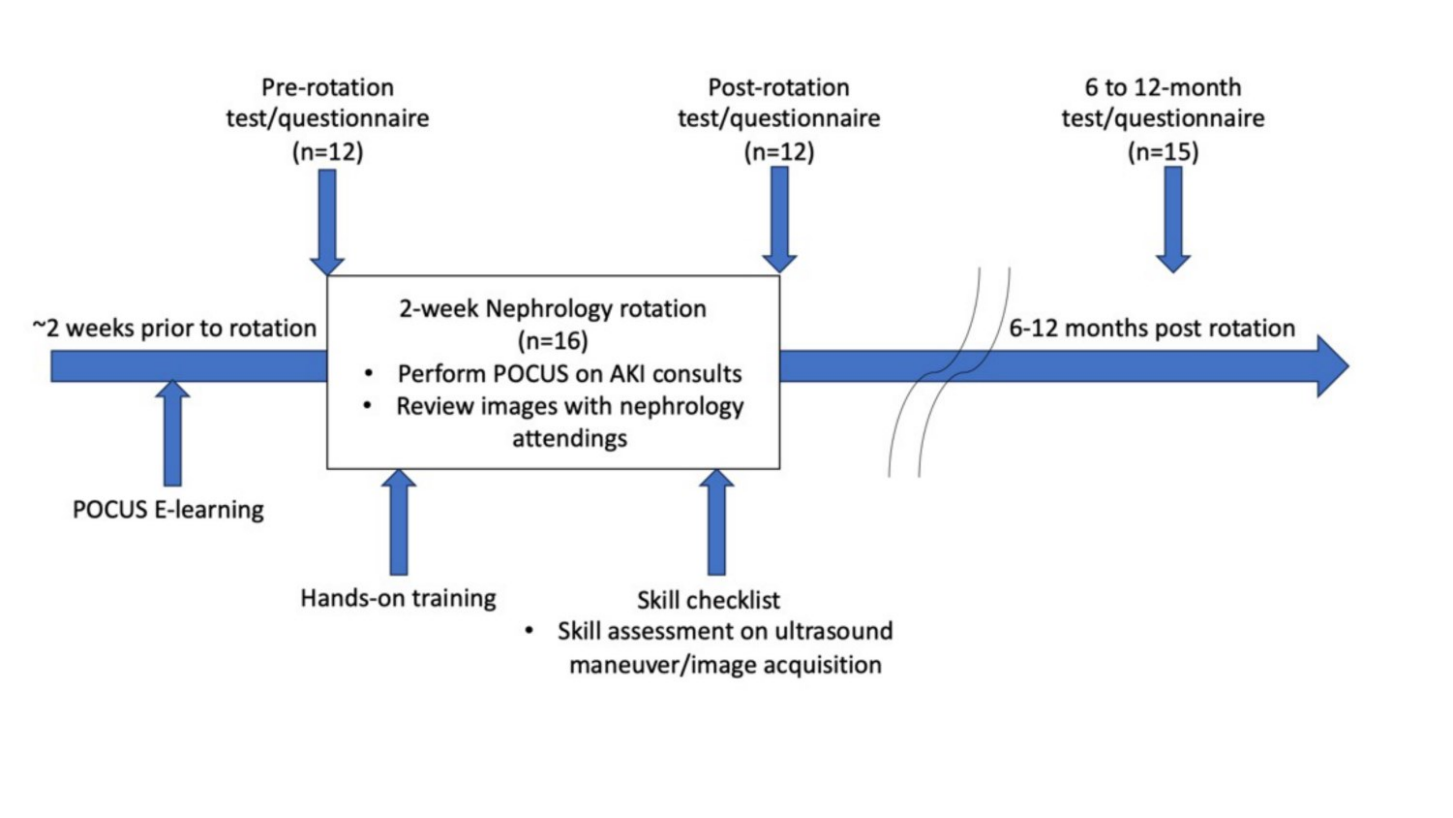
Flowchart of the study

## Results

### Learner Characteristics

Between September 26, 2022 and June 5, 2023, 16 residents completed the nephrology rotation with the embedded POCUS curriculum. Twelve residents completed the pre-rotation questionnaire and test, 12 residents completed the post-rotation questionnaire and test, and 15 residents completed the 6–12-month follow-up questionnaire and test (Figure 1). Among the eight residents who completed the procedure report, 2.25±1.39 POCUS studies were performed during the two-week rotation. The average time from rotation to 6–12-month follow-up testing was 8.29±1.62 months. During the 6-12-month follow-up period, ten residents performed less than five renal POCUS studies, and five residents performed between five and ten POCUS studies. 

### Learner Assessment

Median confidence scores for identifying kidney anatomy, size, hydronephrosis and cysts significantly improved immediately post-rotation and at the 6–12-month follow-up (Table 2). Confidence in estimating bladder volume improved immediately post-rotation but was only partially maintained on follow-up.

Compared to pre-rotation, median knowledge test scores improved immediately post-rotation and at 6–12-month follow-up, but did not achieve statistical significance (pre-rotation: 6.0 [5.0-7.25] vs. post-rotation: 6.5 [5.75-8.0] vs. 6–12-month follow-up: 7.0 [6.0-8.0], p=0.40). We identified a few areas of persistent knowledge gaps immediately post-rotation and on 6–12-month follow-up, specifically in identifying ascites and optimizing depth and machine controls.

Thirteen residents underwent the image acquisition skill test. The median score on the image acquisition skills checklist was 93.1%. All learners were able to correctly use machine controls to optimize their image, and identify the kidney in the short axis, the spleen, the splenorenal recess, and the bladder (Table 3). Only ten (76.9%) learners were able to correctly discriminate the renal cortex, medullary pyramids, and calyces. 

### Comparison between radiology performed and resident-performed POCUS

Thirteen resident-performed renal POCUS studies were saved on the image storage system, out of which five had radiology-performed ultrasound for comparison. Of these, two discrepancies were noted: misidentification of bowel as kidney in a patient with prior nephrectomy and inability to identify the right kidney in one patient.

**Table 2:** Confidence levels and test scores

	**Pre-rotation**	**Post-rotation**	**6-12-months post-rotation**	**p**
**n**	12	12	15	
**Confidence in visualizing medulla and cortex (median [IQR])**	3.00 [1.00, 4.00]	4.00 [4.00, 5.00]	4.00 [3.00, 4.00]	0.007
**Confidence in measuring kidney size (median [IQR])**	2.00 [1.00, 3.00]	4.00 [3.75, 4.25]	4.00 [3.00, 4.00]	0.002
**Confidence in identifying hydronephrosis (median [IQR])**	3.00 [2.00, 3.25]	4.00 [3.00, 4.25]	4.00 [3.00, 4.00]	0.019
**Confidence in identifying nephrolithiasis (median [IQR])**	2.00 [1.00, 2.25]	3.50 [2.75, 4.25]	4.00 [3.00, 4.00]	0.007
**Confidence in identifying kidney cysts (median [IQR])**	2.50 [1.75, 3.00]	4.00 [2.75, 4.25]	4.00 [3.00, 4.00]	0.010
**Confidence in estimating bladder volume (median [IQR])**	2.00 [1.00, 3.00]	4.00 [3.75, 4.25]	3.00 [3.00, 4.00]	<0.001
**Knowledge assessment test score (median [IQR])**	6.00 [5.00, 7.25]	6.50 [5.75, 8.00]	7.00 [6.00, 8.00]	0.400

**Table 3:** Skill checklist scores

Skill checklist item	N (%) correct
Machine set-up downstream so that probe and machine are both in the line of sight of the scanner	13 (100%)
Using low frequency probe	13 (100%)
Dot to left side of screen	13 (100%)
Places probe longitudinally so that probe marker = cephalad	13 (100%)
Obtains longitudinal view of right kidney	11 (84.6%)
Obtains short axis view of right kidney	11 (84.6%)
Identifies liver, hepatorenal recess, and right kidney	12 (92.3%)
Identifies renal cortex, medullary pyramids, and calyces	10 (76.9%)
Uses calipers to measure kidney length from superior to inferior pole	12 (92.3%)
Obtains longitudinal view of left kidney	11 (84.6%)
Obtains short axis view of left kidney	13 (100%)
Identifies spleen, splenorenal recess and left kidney	13 (100%)
Identifies bladder in transverse view	13 (100%)
Scans through bladder to confirm anterior/posterior walls	13 (100%)
Sets gain appropriately: abdominal wall easily distinguished and deeper structures (i.e.. kidney) seen clearly	13 (100%)
Adjusts depth to center organs of interest on screen (i.e.. kidney, hepatorenal recess, bladder)	12 (92.3%)
Records video of each portion of the study	13 (100%)
Interprets findings correctly (normal study vs. specific pathology)	11 (84.6%)
Total	93.1%

## Discussion

We demonstrated that incorporating a Renal POCUS curriculum into an existing nephrology rotation for internal medicine residents is feasible and increases their confidence and image acquisition skills. We noted minimal decay in confidence and knowledge at 6–12-month follow-up. Residents had limited time for deliberate practice which was reflected in their logbooks. Our intervention is unique since it integrated a comprehensive Renal POCUS curriculum for internal medicine residents into a nephrology rotation. The literature on the effectiveness of POCUS curricula for internal medicine residents is highly variable, likely due to the heterogeneity of educational interventions, scope of the objectives, assessment methods, and time to follow-up 10, 11, 12, 13. For example, one study implemented a short lecture followed by a hands-on session and a case-based discussion 10. Another study performed an orientation followed by a longitudinal curriculum over months 12, while another had a workshop followed by an elective where they practiced POCUS and had pre-elective, post-elective and follow-up questionnaires 13. Results on long-term skill and knowledge retention showed conflicting findings amongst these studies focusing on internal medicine residents, which could possibly be explained by the difference in curriculum structure. 

One of the major challenges for our residency program was carving out time in an already compressed and complex curriculum to introduce a new skill that requires both introductory training and deliberate practice, or practice with immediate feedback. Our main goal was to demonstrate the feasibility of integrating this curriculum into the existing workflow of the nephrology consult service while ensuring that residents obtained the basic knowledge and skills of Renal POCUS. This allowed for a resident to immediately apply their knowledge, refine their technical abilities and clinically integrate those skills into the management of patients with AKI.

Although our learners demonstrated an improvement in confidence, we did not show a statistically significant difference in knowledge on the post-test and 6–12-month follow-up test. We suspect that this is due to the small number of learners. However, several other factors may have contributed. First, as the asynchronous, pre-elective didactics were voluntary, there may have been knowledge differences between residents who did and did not watch the lecture. Another factor is that none of the residents were able to meet the goal of completing and reviewing five renal POCUS studies with their nephrology attending during the 2-week rotation. Reinforcement and repetition with immediate feedback from experts are essential to acquiring POCUS knowledge and skills. Thus, a lack of deliberate practice may have contributed to this finding. 

Our study had several limitations. This was a single-center study with residents who were all in their second year of training, which may limit generalizability. Second, our study included only 16 participants with only 12 responses on the pre-and post-rotation surveys and knowledge tests. Prior literature investigating POCUS education had a wide range of participants from 17 to 176, however, the majority did not specifically focus on renal ultrasound 7, 10, 12, 13. Although residents were able to demonstrate basic image acquisition skills for renal POCUS studies, we were not able to statistically evaluate the image quality of each resident due to low portfolio numbers. Lastly, although we were able to evaluate learner confidence, knowledge, and skills, we were not able to assess for any change in their behavior or an effect on patient outcomes. 

## Conclusion

Our renal POCUS curriculum was successfully integrated into an existing two-week nephrology rotation using asynchronous didactic education and hands-on practice during the elective. Resident confidence in renal POCUS examinations improved post-rotation and was sustained on longitudinal follow-up. Residents were able to demonstrate basic image acquisition skills at the end of the rotation. We used Kern’s six-step model for curricular development, which includes an iterative approach based on feedback. Future studies should evaluate curricular improvements, including increasing deliberate practice, as well as the impact on resident behavior and patient outcomes.

## Funding

The authors declare that no funds, grants, or other support were received during the preparation of this manuscript

## Conflict of Interest

The authors have no relevant financial or non-financial interests to disclose

### Authorship Statement

 All authors contributed to the study conception and design. Material preparation, data collection and analysis were performed by Yoshiko Ishisaka, Hong Yu Wang, and Paru Patrawalla. The first draft of the manuscript was written by Yoshiko Ishisaka and all authors commented on previous versions of the manuscript. All authors read and approved the final manuscript.

### Ethics approval

This is an observational study. The Mount Sinai Beth Israel Quality Improvement Committee has confirmed that no ethical approval is required.

Consent to participate: Informed consent was obtained from all individual participants included in the study.

Data availability: The datasets generated during and/or analyzed during the current study are available from the corresponding author on reasonable request.
